# Supplementary material for: Cation-swapped homogeneous nanoparticles in perovskite oxides for high power density
Source: Nat Commun. 2019 Feb 11;10:697. doi: 10.1038/s41467-019-08624-0 (PMC6370853; doi:10.1038/s41467-019-08624-0)
Supplement: Supplementary file 1 — Supplementary Information [file 41467_2019_8624_MOESM1_ESM.docx]

**Supplementary Information**

**Cation Swapped Homogeneous Nanoparticles in Perovskite Oxides for High Power Density**

**Sangwook Joo *et al*.**


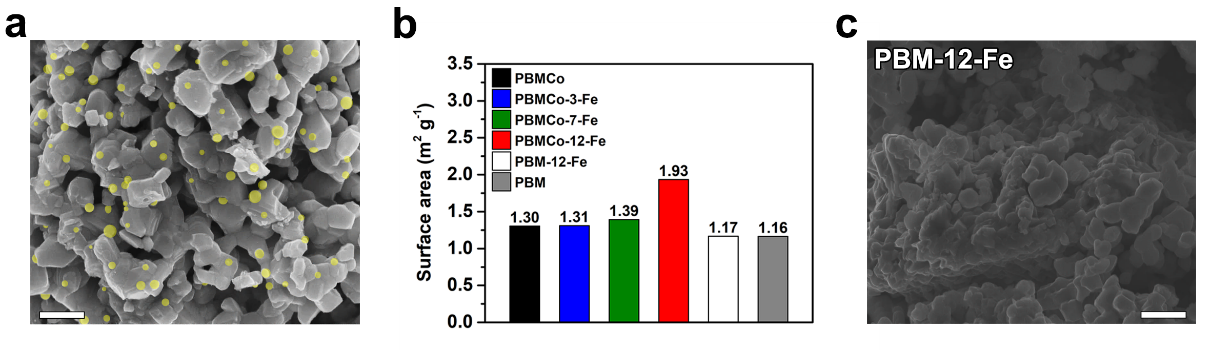


**Supplementray Figure 1.** SEM images and popuration of particles. (a) SEM image of PBMCo-15-Fe; scale bar 500 nm. (b) Comparison of the specific surface area between samples calculated by the BET methods. (c) SEM image of PBM-12-Fe; scale bar 500 nm.


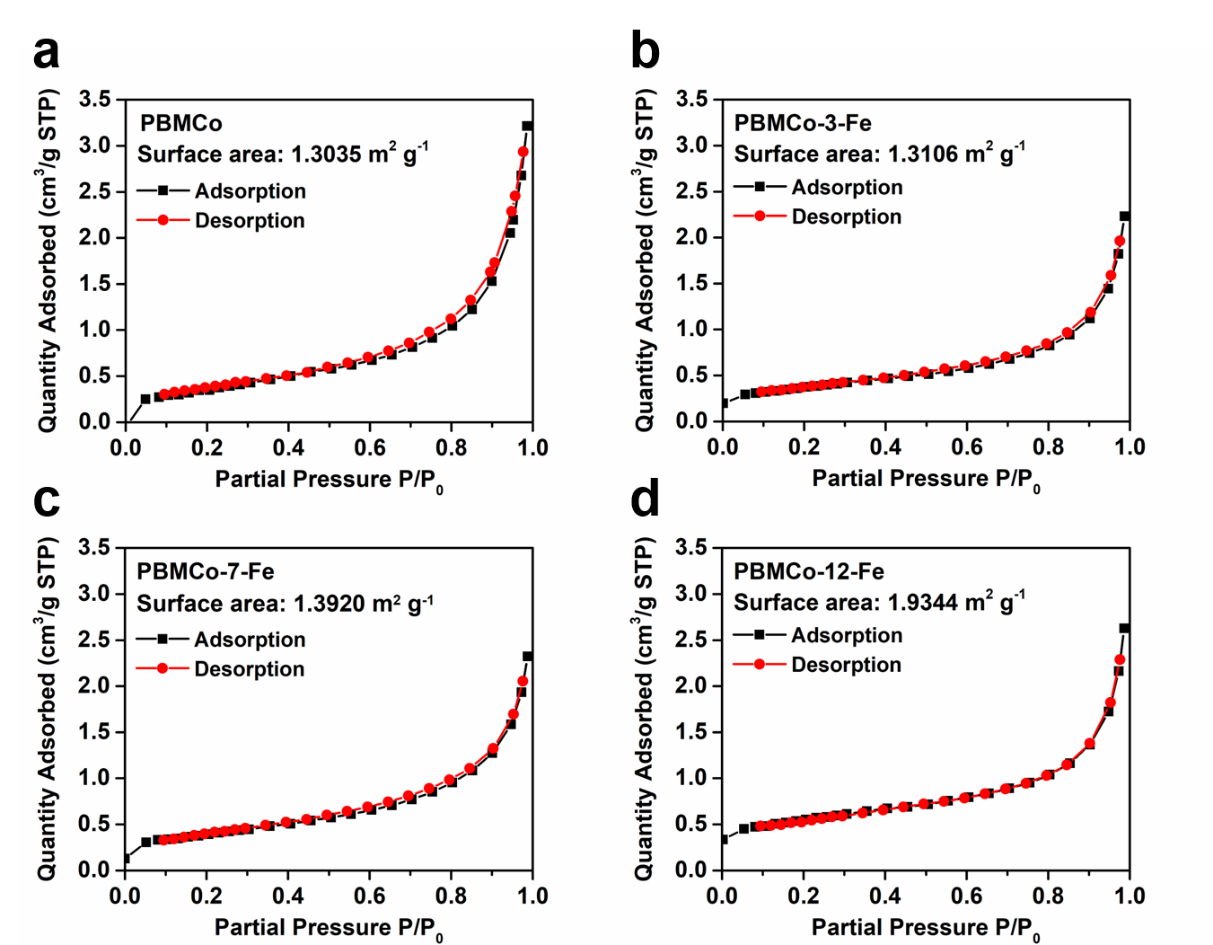


**Supplementray Figure 2.** N_2_ adsorption and desorption isotherms measurement of (a) PBMCo, (b) PBMCo-3-Fe, (c) PBMCo-7-Fe, and (d) PBMCo-12-Fe.


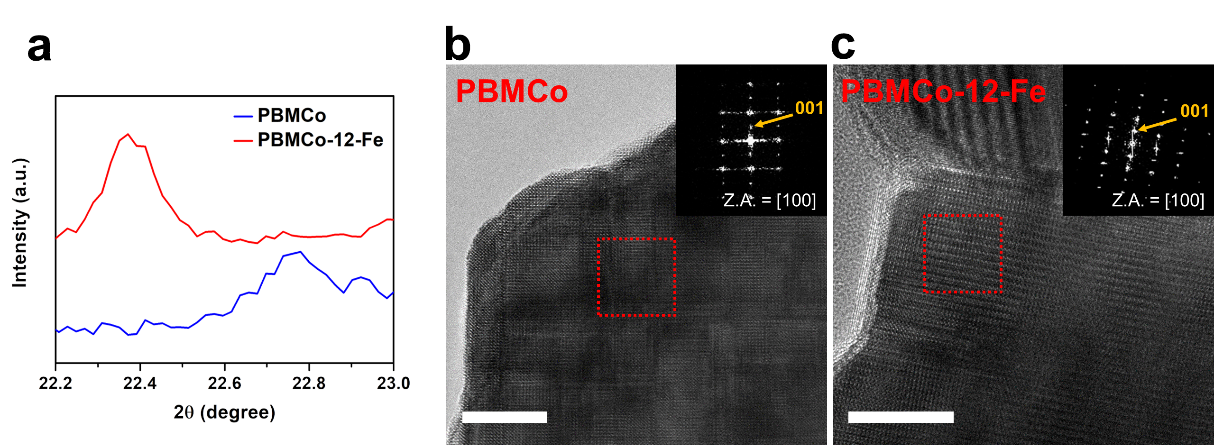


**Supplementray Figure 3.** (a) X-ray diffraction patterns of PBMCo and PBMCo-12-Fe samples around 22 ^o^. HR TEM image of (b) PBMCo and (c) PBMCo-12-Fe samples and the corresponding fast-Fourier transformed pattern with zone axis = [100]; scale bar 10 nm.


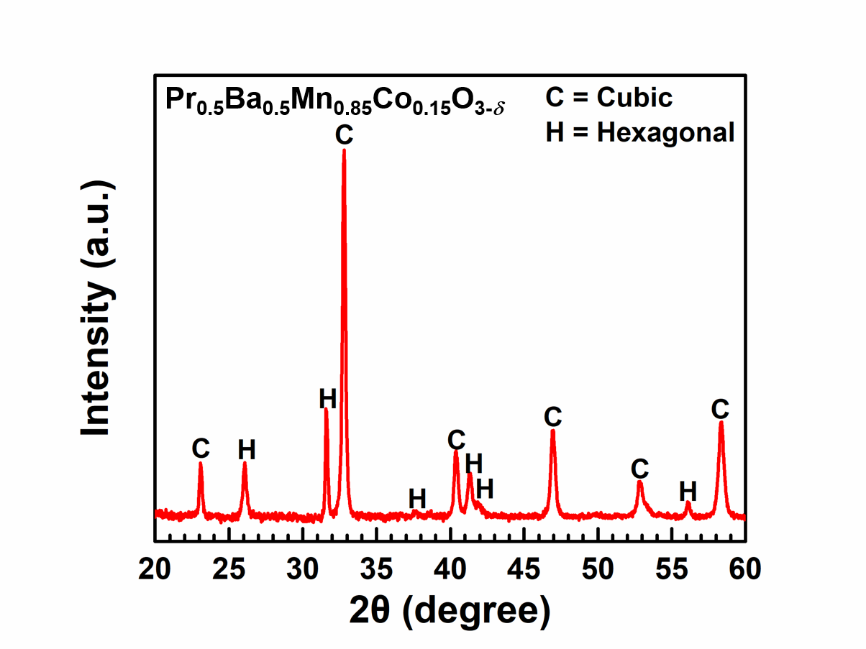


**Supplementray Figure 4.** X-ray diffraction patterns of Pr_0.5_Ba_0.5_Mn_0.85_Co_0.15_O_3-_*_δ_* sintered at 950 ^o^C for 4 h in air.





**Supplementray Figure 5.** X-ray diffraction patterns of PBMCo, PBMCo-3-Fe, PBMCo-7-Fe, and PBMCo-12-Fe samples.


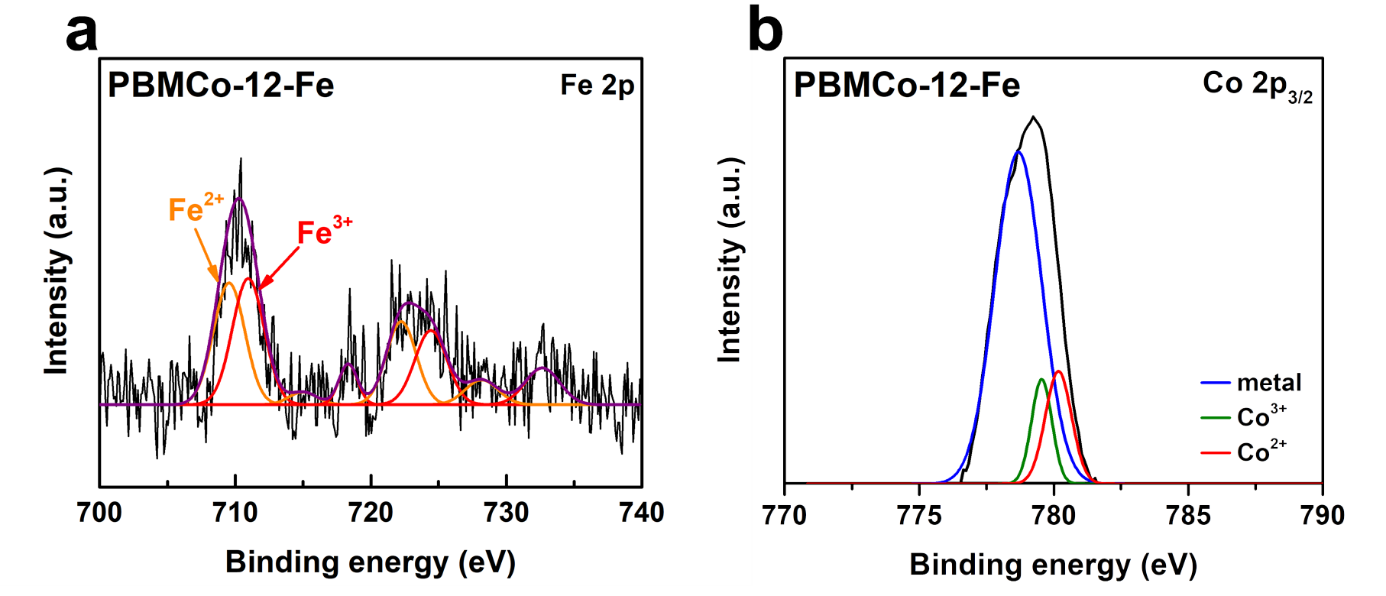


**Supplementray Figure 6.** X-ray photoelectron spectroscopy of (a) Fe 2p for PBMCo-12-Fe and (b) Co 2p_3/2_ for PBMCo-12-Fe.





**Supplementray Figure 7.** I-V curve and power densities of the PBM, PBM-12-Fe, and PBM-12-Co at 800 ^o^C in H_2_ (3% H_2_O).





**Supplementray Figure 8.** I-V curve and power densities of the PBMFe-12-CoFe and PBMCo-12-CoFe at 800 ^o^C in H_2_ (3% H_2_O).


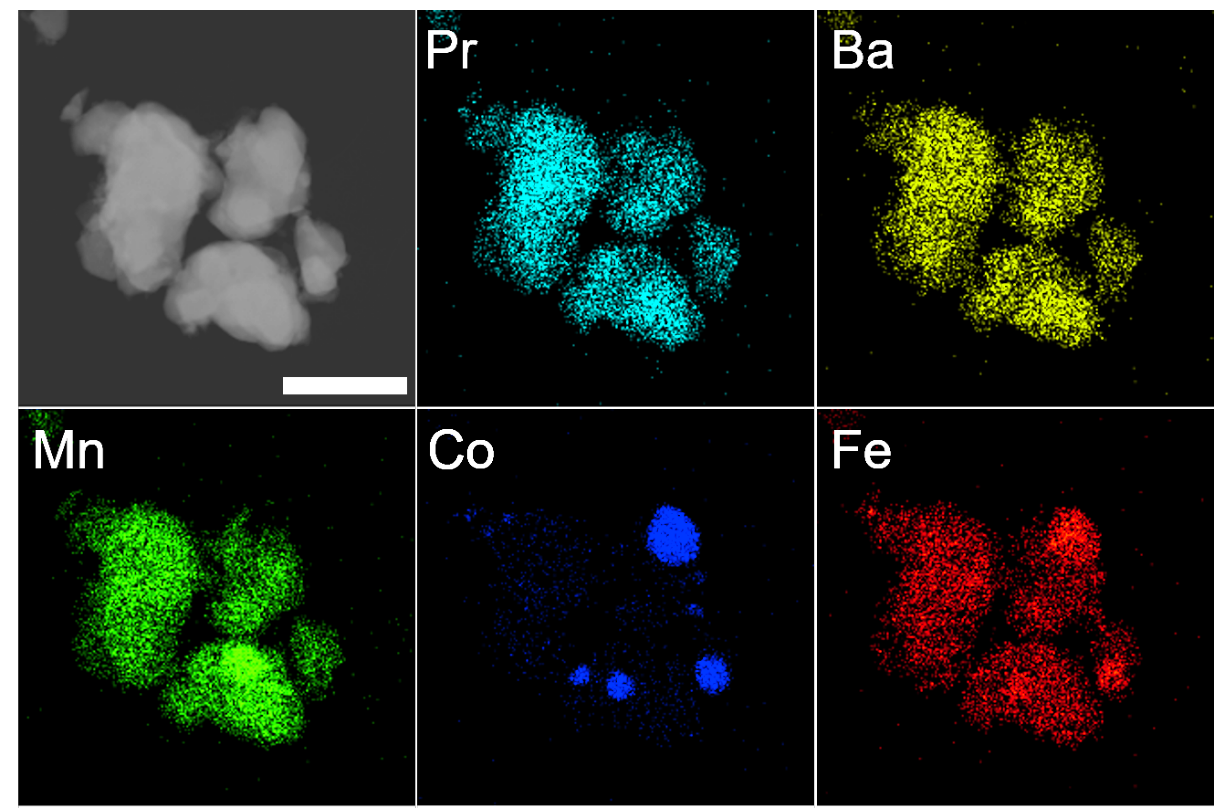


**Supplementray Figure 9.** High-angle annular dark field (HAADF) image of PBMFe-12-CoFe (NPs formed by infiltration) sample with the EDS elemental map of Pr, Ba, Mn, Co, Fe, and O; scale bar 500 nm.





**Supplementray Figure 10.** Impedance spectra of the PBMCo, PBMCo-3-Fe, PBMCo-7-Fe, and PBMCo-12-Fe at 800 ^o^C in H_2_ (3% H_2_O).


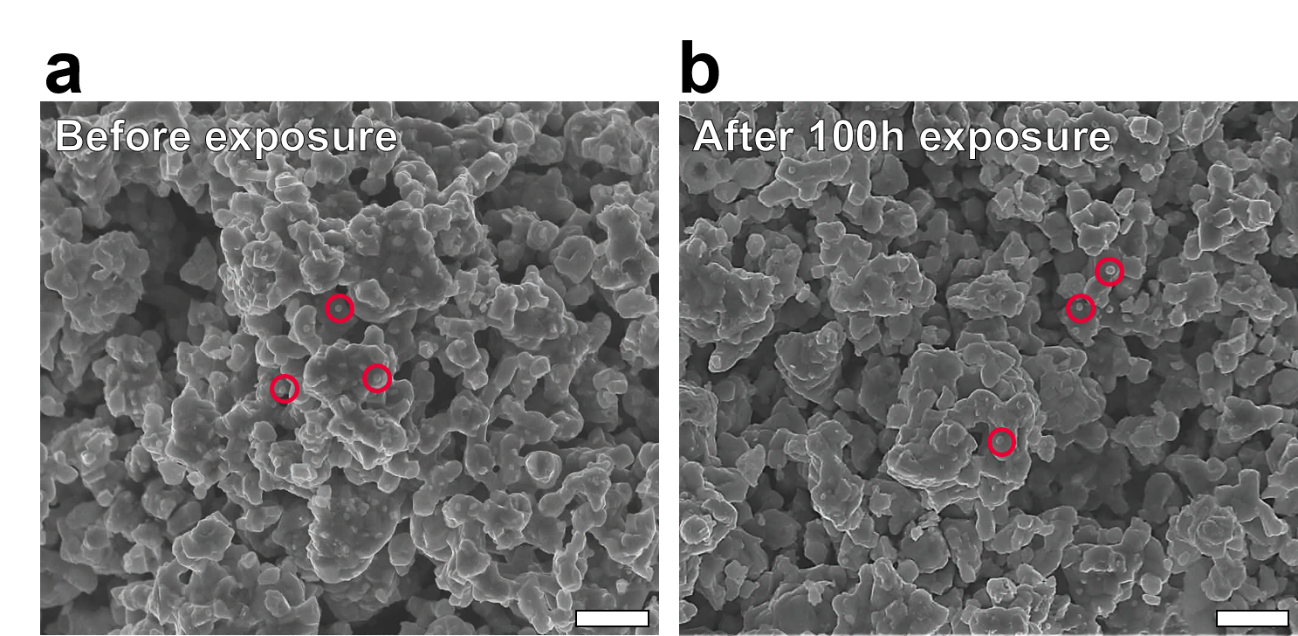


**Supplementray Figure 11.** Comparison in SEM surface morphology of PBMCo-12-Fe before and after exposure to H_2_ (with 3% H_2_O) at 800 ^o^C for 100 hours. The red circles indicate the exsolved nanoparticles; scale bar 500 nm.

**
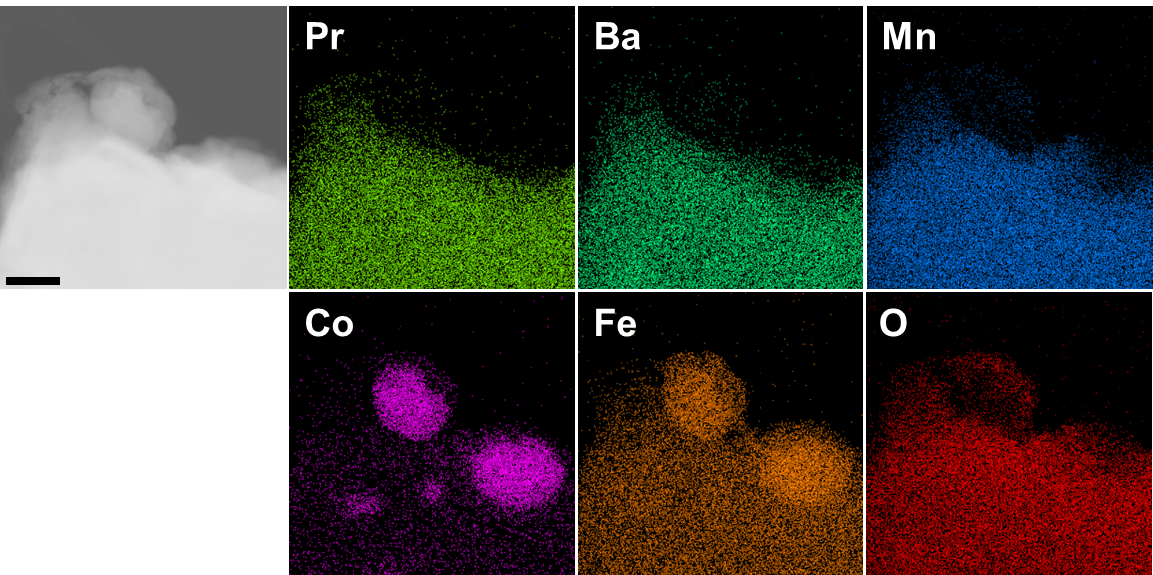
**

**Supplementray Figure 12.** High-angle annular dark field (HAADF) image of PBMCo-12-Fe sample with the EDS elemental map of Pr, Ba, Mn, Co, Fe, and O after DRM test at 900 ^o^C; scale bar 100 nm.


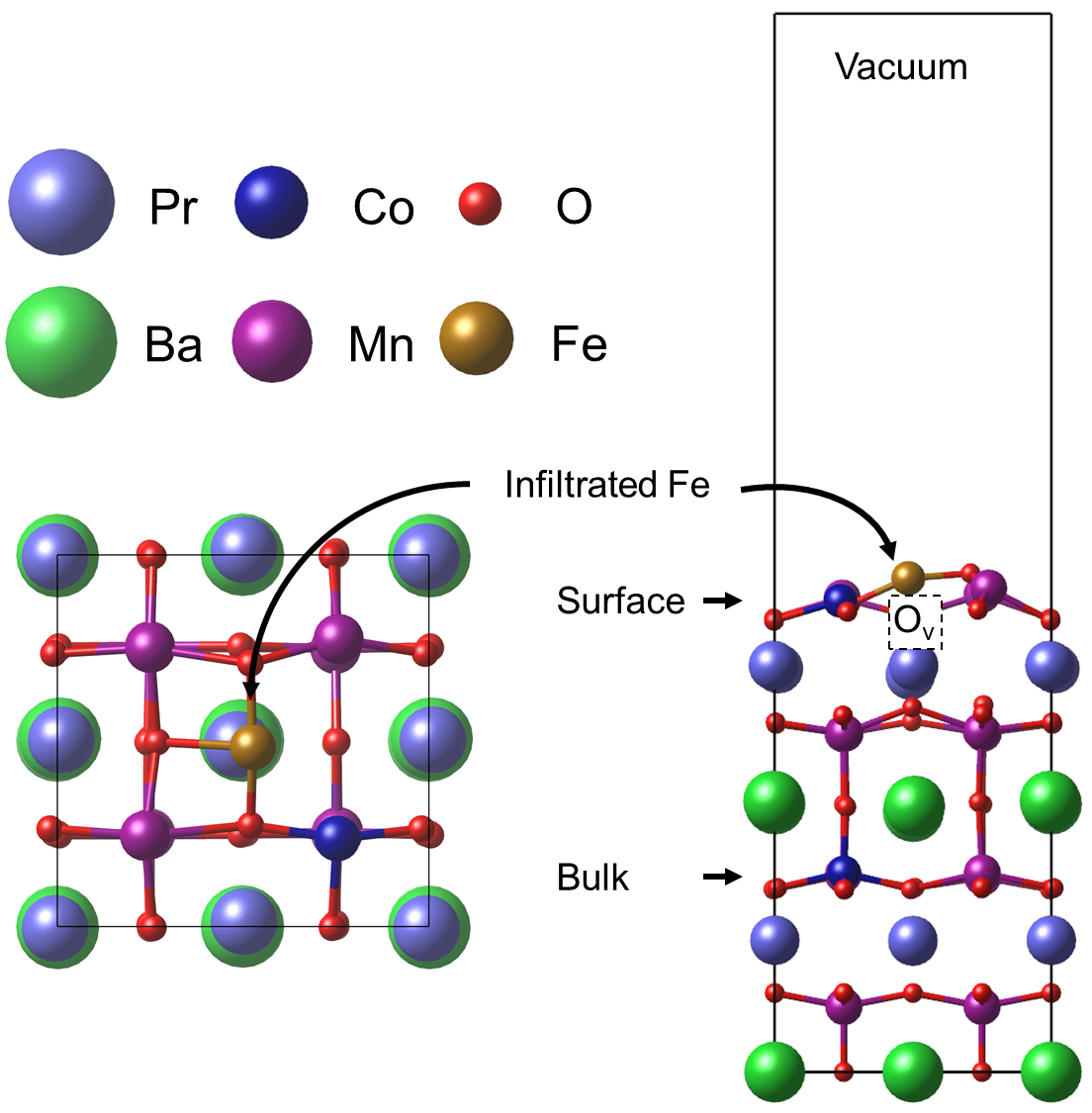


**Supplementray Figure 13.** Top and side views of surface model structures of Fe infiltrated PBMCo used in our DFT calculations. Note that the locations of initial and final states of the segregated Co are assumed to be the first and the fifth layers in our slab model. For the calculations of Fe infiltrated PBMCo, one Fe atom is added on the surface of PBMCo. The oxygen vacancy formation (O_v_) is mostly favorable at a nearest neighbor of surface Co and infiltrated Fe.


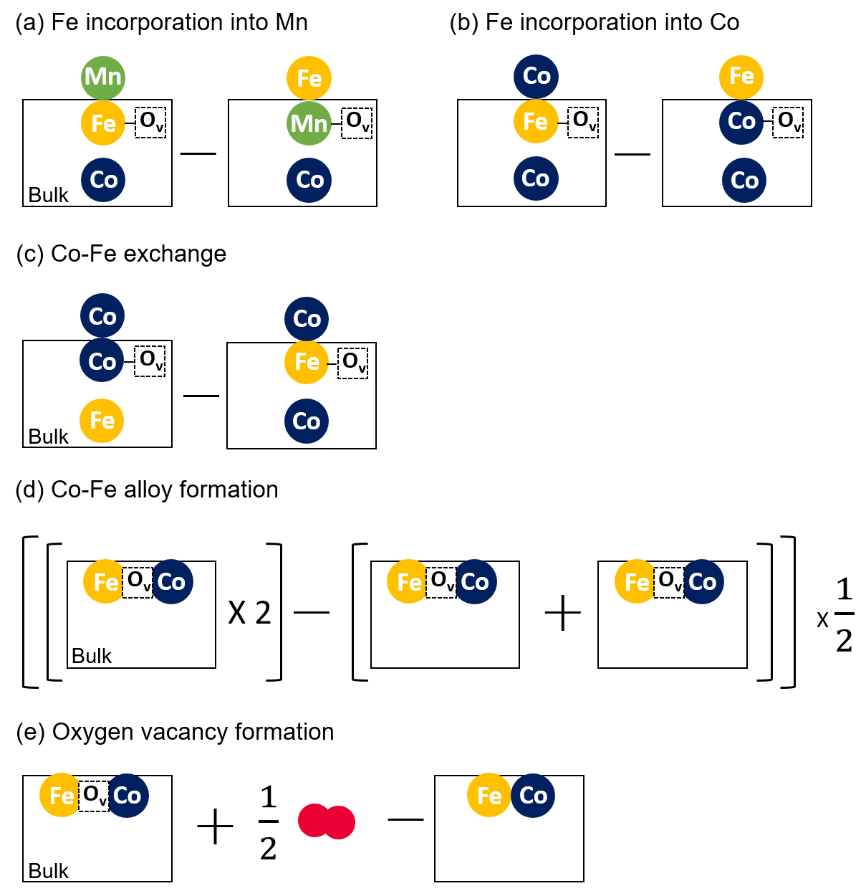


**Supplementray Figure 14.** Schematic illustration for DFT-calculated energetics at each elementary step. The incorporation energy was defined by the total energy difference between the systems where the infiltrated Fe is located on the surface of PBMCo and at the surface B-metal lattice of (a) Mn or (b) Co. (c) The Co-Fe exchange energy was calculated by the total energy difference between the systems before and after the exchange of the location of surface Fe and bulk Co. (d) The alloy formation energy was calculated by the total energy difference between the systems where two different B-site metals are separated and aggregated. (e) The oxygen vacancy formation energy was calculated by total energy difference of the supercells with and without an oxygen vacancy.

**Supplementary Table 1.** The amount of infiltrated Fe in mole percentage.

| **Sample** | **Weight percent of Fe_2_O_3_ infiltrated (%)** | **Mol of Fe_2_O_3_ for the weight percentage to 1 mol of PBMCo** | **Mole of Fe** |
| --- | --- | --- | --- |
| PBMCo-12-Fe | 12 | 0.35 | 0.18 |
| PBMCo-7-Fe | 7 | 0.21 | 0.10 |
| PBMCo-3-Fe | 3 | 0.09 | 0.04 |
| Remarks | *(Weight for 1 mol of PBMCo) = 469.28 g/mol  *(Weight for 1 mol of Fe_2_O_3_) = 159.69 g/mol | | |
